# Next-Generation Dietary Antioxidants in Women’s Reproductive Health: Mechanisms, Reproductive Outcomes, and Therapeutic Potential

**DOI:** 10.3390/antiox15030319

**Published:** 2026-03-03

**Authors:** Md Ataur Rahman, Maroua Jalouli, Mohammed Al-Zharani, Abdel Halim Harrath

**Affiliations:** 1 Department of Oncology, Karmanos Cancer Institute, Wayne State University, Detroit, MI 48201, USA; 2 Department of Biology, College of Science, Imam Mohammad Ibn Saud Islamic University (IMSIU), Riyadh 11623, Saudi Arabia; 3 Zoology Department, College of Science, King Saud University, Riyadh 11451, Saudi Arabia

**Keywords:** dietary antioxidants, oxidative stress, female fertility, reproductive aging, mitochondrial function, redox signaling

## Abstract

Oxidative stress has emerged as a key factor regulating female fertility, reproductive aging, and the development of various gynecologic and pregnancy-associated diseases. While physiological concentrations of reactive oxygen species play a fundamental role in many aspects of normal reproduction such as folliculogenesis, oocyte maturation, implantation, and placental development, abnormal or chronic oxidative stress impairs redox homeostasis and promotes mitochondrial dysfunction, inflammation, DNA damage, and cellular senescence. Recent research interest has shifted toward next-generation dietary antioxidants, including bioactive polyphenols, carotenoids, micronutrients, and nutraceutical combinations with improved bioavailability and molecular targets. These compounds go beyond classical free-radical scavenging activity and modulate a network of redox-sensitive signaling pathways involved in autophagy, apoptosis, endocrine regulation, and immunological balance. In this review, we integrate current mechanistic advances into a cohesive framework that illustrates the regulation of key cellular processes affecting female reproductive physiology by next-generation dietary antioxidants. We also critically evaluate experimental, translational, and clinical data supporting their role in promoting reproductive outcomes, including oocyte quality, ovarian reserve, pregnancy success, and mitigation of age-related reproductive decline. We highlight their potential in the therapeutic intervention of oxidative stress-related conditions such as infertility, polycystic ovary syndrome, endometriosis, early ovarian insufficiency, and menopause-associated disorders. Finally, we discuss the current challenges associated with dosage optimization, bioavailability, long-term safety, and interindividual variability. We conclude by highlighting next-generation dietary antioxidants as a promising, widely available, and non-invasive approach to improve women’s reproductive health and promote fertility throughout their lifespan.

## 1. Introduction

Female reproductive physiology is sustained by a carefully modulated redox environment that promotes physiological signaling and prevents oxidative damage [1]. Reactive oxygen species (ROS) are continuously generated during cellular metabolism and play dual roles in ovarian folliculogenesis, oocyte maturation, steroidogenesis, endometrial remodeling, implantation, and placental development [2]. In physiological amounts, ROS acts as a signaling molecule to coordinate reproductive events. Oxidative stress occurs when antioxidant capacity is overwhelmed and can disrupt cellular homeostasis and result in reproductive dysfunction [3].

Oxidative stress is accumulated over the lifespan of the reproductive system. During the reproductive years, oxidative load can be exacerbated by metabolic stress, inflammation, environmental toxins, endocrine disruptors, and lifestyle factors such as poor nutrition and obesity [4]. With increasing age, mitochondrial dysfunction, decreased antioxidant defenses, and DNA damage accumulation lead to accelerated oocyte aging, aneuploidy, and follicular depletion, culminating in reproductive senescence [5]. Oxidative stress has been closely linked to infertility, diminished ovarian reserve, adverse pregnancy outcomes, and metabolic and inflammatory disorders associated with menopause [6]. Moreover, gynecological conditions like polycystic ovary syndrome, endometriosis, and premature ovarian insufficiency have demonstrated significant oxidative imbalance and inflammatory signaling.

Dietary antioxidants have emerged as an essential line of defense against oxidative stress and have been historically associated with reproductive health benefits [7]. While traditional antioxidants, such as vitamins and trace elements, contribute to redox homeostasis, emerging research shows that next-generation dietary antioxidants offer broader and more targeted biological effects [8]. These include polyphenols, carotenoids, flavonoids, and functional nutraceuticals with improved bioavailability and multitarget activity. In addition to direct ROS scavenging, next-generation antioxidants modulate mitochondrial bioenergetics, inflammatory responses, autophagy, apoptosis, and endocrine signaling, all of which are vital for reproductive fitness and aging [9]. This review provides a comprehensive analysis of next-generation dietary antioxidants in female reproductive health, focusing on their molecular mechanisms, impact on reproductive outcomes, and therapeutic opportunities.

## 2. Next-Generation Dietary Antioxidants in Reproductive Outcomes

**Figure 1:**
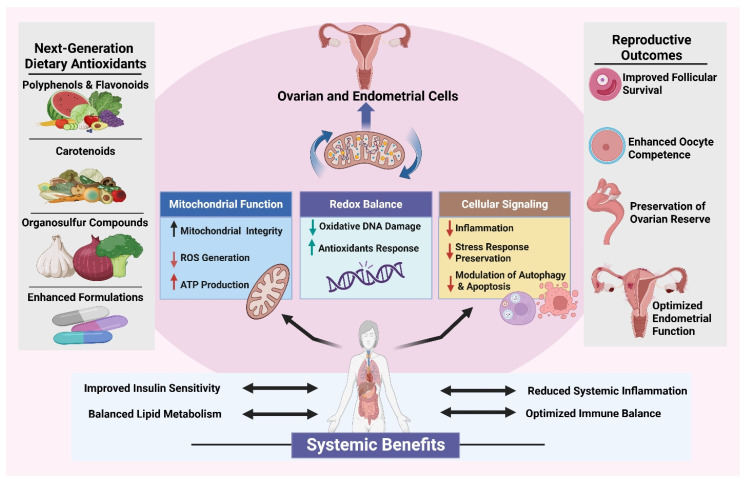
**Next-generation dietary antioxidants and their regulatory roles in reproductive outcomes**. Advanced dietary antioxidants, such as polyphenols, flavonoids, carotenoids, organosulfur compounds, and improved nutraceutical formulations, demonstrate multifaceted regulatory effects on female reproductive organs. These bioactive chemicals affect ovarian and endometrial cells by maintaining mitochondrial integrity, decreasing reactive oxygen species formation, and increasing ATP synthesis, thus facilitating cellular energy metabolism. Simultaneously, they preserve redox equilibrium by reducing oxidative DNA damage and stimulating intrinsic antioxidant response mechanisms. At the signaling level, next-generation antioxidants inhibit inflammatory cascades, regulate stress-response pathways, and optimize autophagy and apoptosis to avert premature follicular loss. In addition to local reproductive benefits, these antioxidants enhance systemic metabolic and immunological balance by improving insulin sensitivity, regulating lipid metabolism, diminishing systemic inflammation, and optimizing immune function. The combined cellular and systemic actions enhance follicular survival, improve oocyte competence, maintain ovarian reserve, and optimize endometrial function, underscoring the therapeutic potential of next-generation dietary antioxidants in achieving positive reproductive outcomes.

Next-generation dietary antioxidants refer to bioactive compounds that are derived from dietary or nutraceutical sources and possess a wide range of regulatory actions beyond their classical free radical scavenging property [10]. The chemicals include polyphenols, flavonoids, carotenoids, organosulfur compounds and newer antioxidant formulations with enhanced bioavailability and tissue targeting. Unlike conventional antioxidants, next-generation chemicals modulate intracellular signaling pathways associated with mitochondrial function, inflammation, autophagy, apoptosis and endocrine signaling [11]. Evidence from research studies suggests that next-generation dietary antioxidants support mitochondrial integrity, reduce oxidative DNA damage and restore redox balance in ovarian and endometrial cells [12]. These chemicals promote follicular survival and oocyte competency by inducing antioxidant response elements and modulating cellular stress responses. Furthermore, their ability to modulate autophagy and apoptosis is particularly relevant for maintaining ovarian reserve and preventing premature follicular loss [13]. Emerging evidence also suggests that next-generation antioxidants influence reproductive outcomes through systemic metabolic and immunological pathways. Improvements in insulin sensitivity, lipid metabolism and inflammatory status may indirectly benefit reproductive function, particularly in cases induced by oxidative stress [1]. These properties make next-generation dietary antioxidants a potential therapeutic option for fertility enhancement and reproductive health interventions. Bioactive products, including polyphenols, flavonoids, carotenoids, organosulfur compounds, and optimized formulations, influence ovarian and endometrial cells to maintain mitochondrial integrity, diminish reactive oxygen species formation, and augment ATP synthesis [14]. These antioxidants preserve redox equilibrium by mitigating oxidative DNA damage and stimulating intrinsic antioxidant responses [15]. At the cellular signaling level, they inhibit inflammation, control stress responses, and influence autophagy and apoptosis [16]. Antioxidant-mediated enhancements in insulin sensitivity, lipid metabolism, immunological equilibrium, and inflammation synergistically facilitate follicular survival, oocyte competence, preservation of ovarian reserve, and optimal endometrial function (Figure 1).

## 3. Women’s Reproductive Health and Oxidative Stress and Redox Signaling

**Figure 2:**
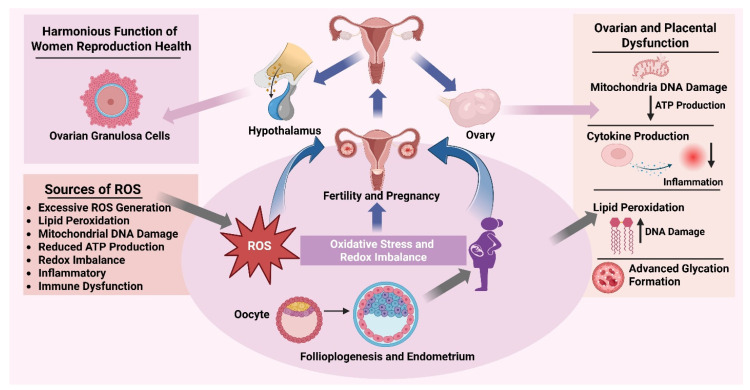
**Women’s reproductive health and oxidative stress and redox signaling.** The pivotal function of redox homeostasis in governing women’s reproductive health via synchronized interactions among the hypothalamic–pituitary–gonadal axis, ovaries, endometrium, and placental tissues. Physiological redox signaling facilitates folliculogenesis, oocyte maturation, ovulation, implantation, fertility, and pregnancy. Excessive formation of reactive oxygen species disrupts equilibrium, resulting in oxidative stress marked by lipid peroxidation, mitochondrial DNA damage, diminished ATP synthesis, inflammatory cytokine release, and immunological dysregulation. The activation of redox-sensitive pathways leads to ovarian and placental malfunction, hinders embryo development, and accelerates reproductive aging, therefore heightening the risk of infertility and pregnancy-related problems.

Women’s reproductive health is the harmonious function of the ovaries, hypothalamic–pituitary–gonadal axis, reproductive tract, and their associated endocrine, metabolic, and immunological systems, which together regulate fertility, pregnancy, and reproductive senescence [17]. Reproductive health requires the exquisite temporal coordination of hormonal signaling, cell homeostasis, and tissue remodeling that allow for folliculogenesis, oocyte maturation, ovulation, implantation, and pregnancy [18]. Disruptions to these events can lead to infertility, adverse pregnancy outcomes, and chronic gynecological and metabolic disorders. Regulated redox signaling facilitates normal ovarian, endometrial, and placental activities crucial for fertility and pregnancy. Conversely, the overproduction of reactive oxygen species induces oxidative stress and redox imbalance, culminating in mitochondrial DNA damage, lipid peroxidation, diminished ATP synthesis, inflammation, and immunological dysfunction. Molecular abnormalities hinder folliculogenesis, oocyte quality, embryo development, and placental function, leading to infertility, negative pregnancy outcomes, and reproductive aging. The schematic emphasizes oxidative stress as a crucial mechanistic connection between impaired redox signaling and reproductive failure (Figure 2).

Oxidative stress is a result of a disruption between the levels of ROS generation and the efficiency of antioxidant defense systems and is defined as a state of cellular homeostasis breakdown [19]. Redox signaling within physiologic limits is essential for normal physiological function in the female reproductive tract, and increased oxidative stress is associated with reproductive failure. ROS are generated within ovarian granulosa cells, oocytes, endometrial cells, and placental tissues, where they are utilized as signaling molecules for folliculogenesis, ovulation, luteinization, and implantation processes [20]. Physiologic levels of low to moderate ROS concentrations promote ovarian steroidogenesis, oocyte meiotic progression, and endometrial receptivity [21]. In contrast, pathologic oxidative stress is associated with lipid peroxidation, protein oxidation, mitochondrial DNA damage, and decreased ATP production [22]. These molecular alterations lead to decreased oocyte quality, impaired embryo development, and reduced placental function. Aging-associated decreases in mitochondrial function result in increased oxidative damage and accelerate follicular depletion and reproductive aging [23]. Redox imbalance is closely linked to inflammatory cues and immune dysregulation in reproductive tissues. Activation of redox-sensitive transcription factors, such as NF-κB and Nrf2, alters cytokine production, antioxidant enzyme expression, and cellular stress response pathways [24]. Dysregulated redox signaling has been linked to infertility, recurrent pregnancy loss, and pregnancy complications, emphasizing the importance of antioxidant defense systems in maintaining reproductive health.

## 4. Female Reproductive Disorders

**Figure 3:**
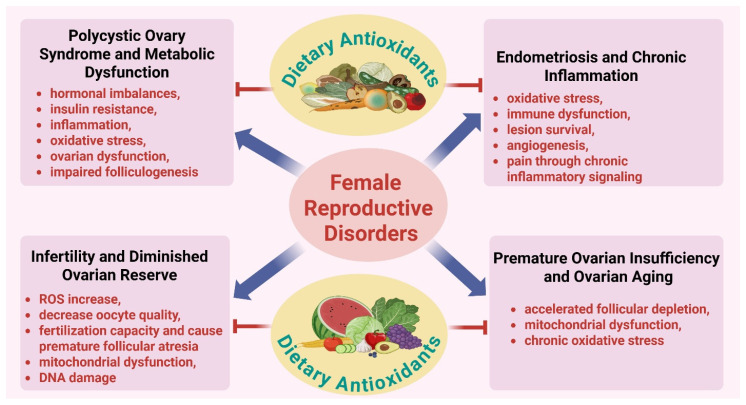
**Role of dietary antioxidants in female reproductive disorders.** Oxidative stress as a prevalent pathogenic factor contributing to significant female reproductive illnesses, such as polycystic ovary syndrome, infertility with reduced ovarian reserve, endometriosis, and early ovarian insufficiency associated with ovarian aging. These diseases are defined by hormonal imbalance, insulin resistance, mitochondrial dysfunction, excessive generation of reactive oxygen species, chronic inflammation, immunological dysregulation, poor folliculogenesis, and rapid follicular depletion. Dietary antioxidants from fruits, vegetables, and bioactive plant chemicals are emphasized as modulatory agents that mitigate redox imbalance, diminish oxidative stress and inflammation, enhance mitochondrial function, and bolster cellular resilience. Antioxidant-based dietary regimens may restore reproductive equilibrium and alleviate the advancement of oxidative stress-induced female reproductive diseases through these pathways.

Oxidative stress is the most shared pathogenic factor in most female reproductive disorders. Antioxidants obtained through diets could potentially prevent redox imbalance, inflammation, and mitochondrial dysfunction, thus improving reproductive performance and slow disease progression in infertile, metabolic, inflammatory, and age-related ovarian conditions (Figure 3).

### 4.1. Infertility and Diminished Ovarian Reserve

Infertility and decreased ovarian reserve are associated with oxidative stress-induced mitochondrial dysfunction, DNA damage and reduced follicular health [25]. High levels of ROS have been shown to decrease oocyte quality, fertilization capacity and cause premature follicular atresia [26]. The supplementation of antioxidants in the diet has the potential to improve the quality of the ovarian microenvironment by restoring redox homeostasis and improving mitochondrial function. Clinical and experimental studies have found that antioxidant intake is correlated with oocyte maturation, embryo quality and improved assisted reproductive technology outcomes [27]. While results are inconsistent, antioxidant-based nutritional interventions have emerged as a potential adjuvant approach to the treatment of oxidative stress-related infertility.

### 4.2. Polycystic Ovary Syndrome and Metabolic Dysfunction

Polycystic ovarian syndrome results from hormonal imbalances alongside insulin resistance and ongoing inflammation with oxidative stress, while nutrition-based treatments can help manage its symptoms. High levels of reactive ROS can contribute to ovarian dysfunction and impaired folliculogenesis [28]. Antioxidant-rich foods could help reduce oxidative and inflammatory stress and improve metabolic health. Phytochemicals found in plant-based foods have shown potential in enhancing insulin sensitivity, modulating inflammatory pathways, and restoring hormonal balance [29]. This could support ovulatory function and reduce metabolic risk in women with PCOS. Antioxidant-based dietary interventions show promise in the holistic management of PCOS.

### 4.3. Endometriosis and Chronic Inflammation

Endometriosis is a chronic inflammatory disease that is strongly associated with oxidative stress and immune dysfunction. Abundant reactive oxygen species promote lesion survival, angiogenesis, and pain through chronic inflammatory signaling [30]. Dietary antioxidants may slow disease progression by mitigating oxidative stress and inflammatory mediator production. Polyphenols and other bioactive compounds have demonstrated potential in reducing the expression of inflammatory cytokines and inhibiting oxidative stress-induced pathways involved in lesion development [31]. Although clinical evidence is limited, antioxidant-based dietary approaches may have a role in complementing standard treatment for endometriosis-related reproductive issues.

### 4.4. Premature Ovarian Insufficiency and Ovarian Aging

Premature ovarian insufficiency and ovarian aging are characterized by accelerated follicular depletion, mitochondrial dysfunction, and chronic oxidative stress. Compromised antioxidant defenses contribute to oocyte apoptosis and diminished ovarian reserve. Dietary antioxidants have the potential to delay ovarian aging by preserving mitochondrial function, preventing oxidative DNA damage, and modulating apoptosis-related signaling pathways [32]. Experimental evidence suggests that antioxidant supplementation improves follicular viability and optimizes ovarian function in conditions of oxidative stress [33]. These findings highlight the potential role of dietary antioxidants as a preventive and supportive strategy for maintaining ovarian function and extending reproductive lifespan.

## 5. Molecular Mechanisms of Dietary Antioxidants in Female Reproduction Management

Nutritional antioxidants can influence female reproduction by impacting mitochondrial homeostasis, redox sensitive signaling, inflammation, autophagy, apoptosis, and hormone homeostasis, which are all important in maintaining cellular redox homeostasis and optimal oocyte quality. Oxidative stress and dysregulation of these processes can contribute to reproductive failure and aging.

### 5.1. Regulation of Mitochondrial Function and Energy Metabolism

Mitochondria are essential for energy metabolism, redox homeostasis and cell survival in the female reproductive system, including oocytes and granulosa cells. Adequate mitochondrial ATP production is critical for folliculogenesis, meiosis progression, fertilization, and early embryonic development [34]. Mitochondrial dysfunction in the context of aging or pathological stress leads to reduced ATP production, increased reactive ROS generation, and accumulation of mitochondrial DNA damage, culminating in impaired oocyte quality and accelerating ovarian aging [5]. Dietary antioxidants play a critical role in maintaining mitochondrial integrity and metabolic homeostasis. Bioactive compounds such as polyphenols, carotenoids and micronutrients boost endogenous antioxidant capacity by upregulating the activity of enzymes like superoxide dismutase, catalase and glutathione peroxidase [35]. These antioxidants help maintain mitochondrial membrane integrity, prevent oxidative damage to mitochondrial respiratory chain complexes, and support efficient electron transport [36]. Recent evidence suggests that next-generation dietary antioxidants activate key metabolic regulators, including AMPK and PGC-1α, leading to mitochondrial biogenesis and improved energy expenditure [37]. The beneficial effects of dietary antioxidants on follicular survival, oocyte maturation and embryonic competence are attributed to their ability to reduce mitochondrial oxidative stress and enhance bioenergetic capacity [38]. These findings are particularly relevant in the context of reproductive aging, highlighting the importance of mitochondrial protection as a key mechanism underlying antioxidant-mediated reproductive benefit. The most recently utilized dietary antioxidants that regulate mitochondrial function and energy metabolism in female reproduction are described in Table 1.

### 5.2. Modulation of Inflammation and Redox-Sensitive Signaling Pathways

Oxidative stress and inflammation are closely intertwined processes. Oxidative stress can induce inflammation, while inflammation can contribute to the production of ROS [49]. This cycle can lead to reproductive failure and aging. Elevated ROS levels can activate redox-sensitive signaling pathways, leading to prolonged inflammation that impairs ovarian and endometrial function [50]. Transcription factors, such as NF-κB, MAPKs, and Nrf2, are involved in regulating inflammatory cytokine production and antioxidant defense mechanisms in reproductive tissues [51]. Antioxidants in the diet can modulate these signaling pathways. They can restore redox balance and suppress chronic inflammatory signaling. Polyphenols and flavonoids can inhibit NF-κB activation, reducing the expression of pro-inflammatory mediators like TNF-α, IL-6, and COX-2 [52]. Concurrently, activation of the Nrf2 pathway can increase the expression of antioxidant response genes, further bolstering cellular defenses [53]. This coordinated regulation helps reduce oxidative inflammation and enhance cellular resilience. In the reproductive context, antioxidant-mediated modulation of redox-sensitive signaling can promote follicular growth, improve endometrial receptivity, and reduce inflammatory stress associated with implantation failure and pregnancy issues [54]. The systemic anti-inflammatory effects can also optimize reproductive function by maintaining metabolic and immunological homeostasis. Table 2 highlights significant dietary antioxidants that influence inflammation and redox-sensitive signaling pathways crucial to female reproduction.

### 5.3. Antioxidant Control of Autophagy and Apoptosis

Autophagy and apoptosis are critical processes that regulate follicular homeostasis, tissue remodeling, and reproductive senescence. Baseline autophagy maintains cellular homeostasis by clearing damaged organelles, while excess apoptosis can contribute to follicular atresia and depletion of ovarian reserve [63]. Oxidative stress can dysregulate the balance between autophagy and apoptosis, leading to pathologic cell death in reproductive tissues [64]. Dietary antioxidants impact autophagy and apoptosis primarily through modulation of intracellular redox status and stress-activated signaling pathways. Antioxidants can promote physiologic autophagic flux by modulating key mediators such as AMPK, mTOR, and Beclin-1, facilitating the removal of damaged mitochondria and protein aggregates (Table 3).

Concurrently, antioxidant function can attenuate oxidative stress-induced apoptotic signaling by blocking mitochondrial cytochrome c release, caspase activation, and pro-apoptotic protein expression [75]. Dietary antioxidants promote follicular survival and preservation of ovarian reserves in part through regulation of autophagy and apoptosis, particularly under conditions of aging or environmental stress. These pathways are relevant to reproductive pathologies characterized by increased cell death or impaired tissue remodeling, highlighting autophagy and apoptosis as important targets for antioxidant-mediated reproductive health (Table 4).

### 5.4. Hormonal Regulation and Endocrine Crosstalk

The integration of ovarian, hypothalamic, pituitary, and peripheral metabolic signals is required for the regulation of female reproduction. Oxidative stress impairs steroidogenesis, steroid hormone receptor signaling, and endocrine feedback loops, causing ovulatory dysfunction, luteal phase defects, and reproductive senescence [86]. In addition, redox dysregulation affects insulin and metabolic hormone signaling, which also has implications for reproductive outcomes. Antioxidants can promote healthy hormonal regulation by protecting steroidogenic tissues from oxidative damage and preserving enzyme function required for the synthesis of estrogen and progesterone [87]. Antioxidants also increase gonadotropin sensitivity in ovarian follicles, promoting follicle maturation and ovulation [88]. Furthermore, the improvements in insulin sensitivity and metabolic signaling that result from antioxidant actions are particularly relevant for conditions like polycystic ovarian syndrome, which involve both endocrine and metabolic disturbances. The cross-talk between reproductive and metabolic endocrine organs is now recognized as having important implications for fertility and reproductive lifespan [89]. Dietary antioxidants promote redox homeostasis, which allows for the integrated signaling between the ovarian, adipose, hepatic, and immune systems [54]. Antioxidant bioactive linked to nutrition are known to affect hormonal regulation and endocrine interactions, encompassing steroidogenesis, insulin signaling, adipokines, estrogen receptor pathways, and endocrine indicators connected to ovarian reserve are presented in Table 5.

### 5.5. Antioxidant Vitamins as Major Contributors to Antioxidant Activity

Antioxidant vitamins are essential regulators of systemic redox homeostasis and are significant donors to endogenous antioxidant defense capacity. In addition to their traditional function as free radical scavengers, recent findings indicate that vitamins A, C, D, and E modulate signaling pathways associated with autophagy, mitochondrial activity, and inflammatory regulation, thereby impacting the biology of aging and longevity [100].

Vitamin C (ascorbic acid) is a powerful antioxidant that dissolves in water and neutralizes reactive oxygen species [101]. It also helps other antioxidants, like vitamin E, work better. Oxidative stress significantly impedes autophagic flow by inducing oxidative modifications in autophagy-related proteins and lysosomal enzymes [102]. Vitamin C indirectly stabilizes autophagy machinery and protects lysosomal function by restoring redox equilibrium [103]. Experimental evidence indicates that vitamin C may influence AMPK activity and enhance mitochondrial turnover during oxidative stress, while human data regarding direct autophagy biomarkers are still scarce [104].

Vitamin E (α-tocopherol) is a lipid-soluble antioxidant that stops lipid peroxidation in cellular and lysosomal membranes. Lipid peroxidation compromises lysosomal integrity and hinders autophagic degradation [105]. Supplementation with vitamin E has demonstrated the capacity to safeguard membrane integrity and mitigate oxidative damage linked to inflammation, mechanisms that indirectly sustain autophagy-lysosome functionality during the aging process [106].

Vitamin D, historically recognized for its role in calcium metabolism, also governs transcriptional processes associated with immunological regulation and autophagy [107]. The activation of the vitamin D receptor is linked to the overexpression of autophagy-related genes including Beclin-1 and LC3 in immune cells and epithelial tissues [108]. This suggests that vitamin D may help keep cells in balance and slow down the aging process.

Vitamin A and its derivatives (retinoids) affect how cells differentiate, how they respond to oxidative stress, and how the immune system works [109]. Retinoic acid signaling has demonstrated interactions with mTOR-related pathways and cellular stress responses, suggesting possible crosstalk with autophagic control, but findings are context-dependent [110].

Antioxidant vitamins collectively modulate aging by stabilizing redox-sensitive autophagy pathways, maintaining lysosomal functionality, and mitigating chronic inflammation [111]. However, although there is a lot of molecular evidence, more well-designed clinical studies are needed to explicitly prove their significance in human autophagic flux modulation and longevity effects.

## 6. Dietary Antioxidants and Delayed Reproductive Aging: Implications for Ovarian Longevity

Reproductive aging is marked by a gradual deterioration in ovarian reserve, oocyte quality, and hormonal equilibrium, culminating in diminished fertility and the onset of menopause. Oxidative stress, mitochondrial dysfunction, persistent low-grade inflammation, and compromised autophagy are significant factors in ovarian aging. Oocytes are especially susceptible to oxidative damage owing to their elevated metabolic activity and extended halt in meiosis. The buildup of reactive oxygen species can damage DNA, shorten telomeres, cause problems with mitochondria, and make granulosa cells work less well, which speeds up the loss of follicles.

Antioxidants from fruits, vegetables, whole grains, and plant-based meals may help fight these processes by restoring redox equilibrium and keeping mitochondria healthy. Resveratrol and quercetin are two polyphenols that have been shown to stimulate AMPK and SIRT1 signaling, which are pathways that are connected to improved autophagy and the creation of new mitochondria. Restoring autophagic flow in ovarian tissue may aid in the elimination of damaged mitochondria and protein aggregates, thus maintaining oocyte competence. Carotenoids and antioxidant vitamins, such as vitamins C and E, help stabilize membranes and protect against lipid peroxidation. This is important for keeping granulosa cells working and the follicular milieu stable.

Recent preclinical findings indicate that the regulation of oxidative stress and autophagy pathways may postpone follicular atresia and preserve ovarian reserve. While human clinical data is still limited and needs more testing, regularly eating foods high in antioxidants may be a safe and long-lasting way to help the ovaries stay healthy. Combining dietary antioxidants with individualized nutrition strategies shows potential for enhancing reproductive health and prolonging reproductive longevity.

## 7. Clinical and Translational Evidence Supporting Dietary Antioxidants

Clinical and translational evidence increasingly supports the role of dietary antioxidants in improving female reproductive health by mitigating oxidative stress, inflammation, and metabolic abnormalities. Observational studies have consistently reported significant associations between antioxidant-rich diets and improved fertility parameters, ovarian reserve markers, and pregnancy outcomes [112]. Higher intakes of fruits, vegetables, and polyphenol-rich foods have been linked to better oocyte quality, increased endometrial receptivity, and reduced risk of reproductive disorders [113]. Antioxidant supplements in interventional studies have yielded variable but promising results. In the context of infertility and assisted reproductive technologies, antioxidants have been found to improve oocyte maturation, embryo quality, and implantation rates, particularly in women with elevated oxidative stress levels [114]. In conditions like polycystic ovarian syndrome and endometriosis, where metabolic and inflammatory dysregulation are common, antioxidant therapies have shown promise in reducing inflammatory markers, improving insulin sensitivity, and restoring hormonal balance. Clinical evidence also suggests a potential role for dietary antioxidants in mitigating age-related reproductive decline and metabolic dysfunction associated with menopause (Table 6). Despite these encouraging findings, heterogeneity in study designs, antioxidants used, dosages, and treatment durations limit direct comparisons and definitive conclusions. The bioavailability, long-term safety, and interindividual variability in antioxidant responses remain critical areas of concern. Precision nutrition approaches that consider biomarkers of oxidative stress and metabolic health may improve treatment efficacy. Translational evidence positions dietary antioxidants as a widely accessible, non-invasive adjunctive strategy in reproductive medicine [115]. Rigorously designed, large-scale clinical trials are warranted to determine optimal antioxidant regimens and validate their long-term benefits in women’s reproductive health.

## 8. Regulatory Status and Clinical Approval of Antioxidant Compounds

There are a lot of antioxidant chemicals that people can buy, but their regulatory classification and approval status are very different. The Dietary Supplement Health and Education Act (DSHEA) of 1994 says that most antioxidant vitamins and bioactive natural compounds are sold as dietary supplements in the United States [130]. In this system, the U.S. Food and Drug Administration (FDA) does not need to approve supplements before they can be sold, but the companies that make them oversee the process of making sure they are safe and properly labeled [131]. As a result, most of the polyphenols, flavonoids, alkaloids, terpenoids, and carotenoids that are talked about in this review are not FDA-approved medications for treating or preventing aging, neurological diseases, or other conditions that come with age. The FDA has nevertheless approved several antioxidant-related chemicals for certain medical uses. For instance, prescription forms of vitamin D are approved for treating illnesses caused by a lack of vitamin D, and certain retinoids, which are vitamin A derivatives, are approved for skin and cancer issues [132]. These approvals are only for certain uses and do not include claims on anti-aging or changing autophagy. Likewise, certain omega-3 fatty acid formulations possess FDA approval for the management of hypertriglyceridemia but lack approval as therapeutic agents for aging [132]. It is important to remember that regulatory approval for a certain use does not automatically support larger claims like longevity or improving autophagy. So, it is important to make a clear distinction between the status of supplements, FDA approval for certain uses, and experimental anti-aging treatments. This distinction aids in averting the overinterpretation of mechanistic data and facilitates the prudent clinical application of antioxidant-based therapies in aging research.

## 9. Dietary Antioxidants as Specific Food Sources and Their Health Benefits for Public Translation

Diet constitutes the principal and most physiologically significant source of antioxidant bioactive chemicals. Whole meals contain a wide range of polyphenols, carotenoids, vitamins, minerals, fiber, and synergistic phytochemicals that work together to affect redox balance and autophagy regulation [133]. This is different from taking supplements on their own. Focusing on eating foods high in natural antioxidants is a good way to promote healthy aging and help cells stay in balance through autophagy [134]. Berries, pomegranate, grapes, and citrus fruits are all high in polyphenols, flavonoids, and vitamin C [135]. Resveratrol, quercetin, anthocyanins, and ellagitannins are some of the compounds that have been related to AMPK activation, mTOR regulation, and better mitochondrial function [136]. Carrots, tomatoes, leafy greens, and cruciferous vegetables all provide carotenoids like lutein, beta-carotene, and lycopene [137]. These carotenoids help to keep redox-sensitive autophagy pathways open by protecting lysosomal membranes and autophagy-related proteins from oxidative damage. Whole grains, legumes, nuts, and seeds provide polyphenols, vitamin E, spermidine, and trace minerals that help cells to adapt to stress and make the body more resilient [138]. Fermented foods and high-fiber diets also change the makeup of gut microbiota, which increases the synthesis of bioactive metabolites such urolithin A [139]. This metabolite has been linked to mitophagy activation and better muscular performance in older people. Marine sources, such as fatty fish and seaweed, offer carotenoids and omega-3 fatty acids that mitigate inflammation, thus indirectly promoting autophagic equilibrium [140].

Dietary patterns, exemplified by the Mediterranean diet, that emphasize the high consumption of fruits, vegetables, whole grains, legumes, and olive oil, as well as a moderate intake of nuts and fish, consistently correlate with a diminished risk of neurodegeneration, cardiovascular disease, and metabolic disorders [141]. These advantages are believed to stem from the continuous consumption of various antioxidant chemicals that enhance redox balance, mitochondrial function, and controlled autophagy. Public translation should focus on diversity and moderation instead of high-dose supplementation [142]. Encouraging people to eat plant-based meals that are minimally processed on a regular basis can help with redox signaling and adaptive autophagy in the body. This food-based method fits with existing nutrition guidelines and is a safe, long-term way to improve health span and encourage healthy aging in the general population.

## 10. Dietary Antioxidants and Assisted Reproductive Technology (ART) Outcomes

Oxidative stress is a major factor in reproductive aging and lower fertility, especially in women who are using Assisted Reproductive Technology (ART) [143]. Increased reactive oxygen species in follicular fluid and the reproductive tract can hinder oocyte maturation, fertilization capability, embryo development, and implantation efficacy [144]. As a result, dietary antioxidants have been popular as helpful treatments in ART settings. Researchers have investigated several antioxidant chemicals to see if they can boost ART outcomes [145]. Coenzyme Q10, a mitochondrial cofactor exhibiting antioxidant characteristics, has been linked to enhanced oocyte quality and ovarian response in women with reduced ovarian reserve [146]. Vitamins C and E have been assessed for their efficacy in mitigating oxidative stress in follicular settings; nevertheless, clinical outcomes are inconsistent and frequently contingent upon dosage and patient-specific factors [147]. Melatonin, a strong antioxidant found in follicular fluid, may help to improve the maturation of oocytes and the rates of fertilization [148]. Dietary patterns high in polyphenols may enhance redox balance and mitochondrial activity, both of which are essential during folliculogenesis and early embryogenesis [113]. Although increasing evidence is encouraging, studies differ in design, sample size, and supplementation techniques. Consequently, rigorously controlled randomized clinical trials are essential to formulate uniform guidelines and elucidate which antioxidants, dosages, and patient demographics yield the most significant advantages in ART settings.

## 11. Limitations, Challenges, and Knowledge Gaps

Despite accumulating evidence supporting the positive effects of dietary antioxidants on women’s reproductive health, several limitations and challenges must be acknowledged and addressed. One prominent issue is the substantial heterogeneity present across the existing literature. Variations in study designs, populations, sources of antioxidants, dosage regimens, treatment duration, and outcome measures make direct comparisons and generalizations difficult, limiting the strength of conclusions [149]. Additionally, several therapeutic studies involve small sample sizes or short treatment durations, compromising statistical power and long-term clinical relevance. Another critical challenge lies in the bioavailability and metabolism of antioxidants. The absorption, distribution, and physiological activity of dietary antioxidants can be significantly influenced by the specific chemical form of the antioxidant, the food matrix in which it is consumed, the composition of the gut microbiota, and individual metabolic factors [150]. Therefore, circulating antioxidant levels may not accurately reflect tissue-specific effects in reproductive organs. Furthermore, excessive or unbalanced antioxidant supplementation can disrupt physiological redox signaling pathways, which are essential for normal reproductive function, highlighting the importance of appropriate dosing.

Interindividual variability is another significant gap in knowledge. Factors such as genetic predisposition, age, metabolic status, environmental influences, and baseline oxidative stress levels can all influence individual responses to antioxidant interventions [151]. However, most current research does not stratify participants based on these factors, limiting the applicability of findings to personalized reproductive healthcare. In addition, most studies focus on individual antioxidants, rather than overall dietary patterns or combinations, which may be more representative of real-world consumption. Mechanistic gaps also remain, particularly concerning the specific molecular targets of next-generation dietary antioxidants in human reproductive tissues. A significant portion of mechanistic knowledge is derived from in vitro or animal studies, with limited validation in clinical settings. Furthermore, there is a paucity of long-term safety data, particularly in the context of pregnancy or prolonged supplementation. Addressing these limitations and challenges will require well-designed, large-scale clinical trials incorporating biomarker-driven approaches, standardized outcome measures, and precision nutrition strategies. These efforts will be essential to fully elucidate the therapeutic potential of dietary antioxidants in women’s reproductive health.

Antioxidants are generally thought to be good for fighting oxidative stress and aging, but taking too many of them might have bad effects. Reactive oxygen species are not merely deleterious consequences; they also serve as crucial signaling molecules that modulate physiological processes, including autophagy, mitochondrial biogenesis, immunological responses, and cellular tolerance to stress. Excessive inhibition of oxidative signaling by elevated antioxidant consumption may hinder redox-sensitive pathways, including AMPK activation and mitohormesis, consequently compromising advantageous stress adaptation mechanisms. Extensive clinical research indicates that prolonged high-dose supplementation of certain antioxidant vitamins, notably vitamins A and E, may provide neutral or even detrimental effects in specific groups, including an elevated risk of mortality or various malignancies [152]. Antioxidants that dissolve in fat may build up in tissues over time, which raises questions about their safety and toxicity in the long term. In addition, too much exposure to antioxidants may interfere with the body’s natural adaptations to exercise and immunological signaling, which could negate the health benefits that come from within it [153]. So, it is important to keep the body’s redox equilibrium instead of loading it up with random antioxidants. Future interventions must emphasize suitable dose, personalized techniques, and the assessment of long-term safety.

## 12. Future Perspectives and Emerging Directions

Optimizing the role of dietary antioxidants in women’s reproductive health will require a shift from the current paradigm to one that is more precise, integrative, and innovative. Precision nutrition approaches that tailor antioxidant interventions based on individual oxidative stress levels, metabolic status, genetic variations, and specific reproductive stages may be the future of antioxidant therapy in women’s health. Validated biomarkers for redox status, mitochondrial function, and inflammation can enable more accurate patient stratification and improved treatment outcomes.

Emerging interest in next-generation dietary antioxidants and bioavailable functional foods offers new opportunities for reproductive health optimization. Advances in delivery systems, such as nanoformulations and synergistic combinations of antioxidants, could improve tissue targeting and biological activity while minimizing potential side effects [154]. Furthermore, food pattern-based interventions instead of single-agent supplementation may more accurately reflect physiological exposures and promote sustainable reproductive benefits. The integration of dietary antioxidants with lifestyle modifications, reproductive medicine, and metabolic health management represents an exciting frontier. A holistic approach that targets nutrition, exercise, and environmental factors may have additive or synergistic effects on fertility and reproductive aging. Additionally, strengthening translational research efforts to bridge the gap between mechanistic studies and clinical outcomes will support evidence-based guidelines. Longitudinal and life-course research is needed to evaluate the long-term effects of antioxidant consumption on fertility trends, reproductive aging, and menopause-related health issues. These emerging areas will collectively position dietary antioxidants as a scientifically validated, personalized, and effective solution for optimizing women’s reproductive health across the lifespan.

## 13. Conclusions

Antioxidants present in foods are necessary to maintain redox homeostasis and for female reproductive health over the entire lifespan. Emerging dietary antioxidants can offer multiple lines of defense against oxidative stress-related reproductive damage through their effects on mitochondria, inflammation, autophagy, apoptosis, and hormone balance. Preclinical and clinical studies suggest that dietary interventions with antioxidants may improve fertility outcomes, delay reproductive aging, and mitigate progression of common reproductive disorders. The individual variability, bioavailability, and study design differences highlight the need for precision nutrition approaches. Incorporating mechanistic understanding, biomarker-targeted therapies, and well-designed clinical trials in the future will be important for translating dietary antioxidants into personalized interventions for female reproductive health.

## Figures and Tables

**Table 1:** Dietary antioxidants modulating mitochondrial function and energy metabolism in female reproduction.

Dietary Antioxidant	Major Dietary Source	Key Mitochondrial Targets	Mechanism of Action	Reproductive Relevance	Study Type/Model (In Vitro/In Vivo)	Human Clinical/ Epidemiological Investigations	Ref.
Resveratrol	Grapes, berries, red wine	AMPK, PGC-1α, SIRT1	Enhances mitochondrial biogenesis and ATP production, reduces ROS	Improves oocyte quality and delays ovarian aging	In vitro (granulosa cells), In vivo (animal ovarian aging models)	Limited clinical evidence, small human fertility studies	[39]
Quercetin	Onions, apples, citrus fruits	Mitochondrial membrane potential	Stabilizes mitochondrial membranes, reduces oxidative damage	Supports follicular survival and oocyte competence	Primarily in vitro, some animal models	Limited direct clinical evidence	[40]
Epigallocatechin gallate	Green tea	Electron transport chain complexes	Improves mitochondrial efficiency and reduces ROS generation	Enhances oocyte maturation and embryo development	In vitro (oocyte models), In vivo (rodent studies)	Limited epidemiological data	[41]
Curcumin	Turmeric	AMPK, mitochondrial ROS	Promotes mitochondrial biogenesis and antioxidant enzyme expression	Protects ovarian reserve and improves energy metabolism	In vitro and in vivo models	No standardized clinical ART trials	[42]
Coenzyme Q10	Meat, fish, whole grains	Electron transport chain (Complex I–III)	Facilitates electron transport and ATP synthesis	Improves oocyte mitochondrial function and fertilization outcomes	In vivo animal models	Randomized clinical trials in women with diminished ovarian reserve	[43]
Melatonin	Fruits, grains, endogenous synthesis	Mitochondrial permeability transition pore	Preserves mitochondrial integrity and reduces oxidative stress	Enhances oocyte quality and embryo viability	In vitro and in vivo models	Clinical trials in IVF patients	[44]
Lycopene	Tomatoes, watermelon	Mitochondrial lipid membranes	Prevents lipid peroxidation and mitochondrial damage	Supports ovarian function under oxidative stress	Primarily animal studies	Limited epidemiological data	[45]
Astaxanthin	Algae, seafood	Mitochondrial ROS scavenging	Protects mitochondrial membranes and improves bioenergetics	Preserves follicular integrity and reduces aging-related decline	In vitro and animal models	Emerging clinical observations, limited RCT data	[46]
Alpha-lipoic acid	Spinach, broccoli	Mitochondrial redox enzymes	Regenerates antioxidants and improves mitochondrial metabolism	Improves ovarian mitochondrial efficiency	In vitro and animal models	Some small clinical trials in metabolic and fertility contexts	[47]
Omega-3 fatty acids	Fish oil, flaxseed	Mitochondrial membrane fluidity	Enhances mitochondrial membrane function and energy metabolism	Supports follicular development and metabolic balance	In vitro and animal models	Epidemiological and some interventional reproductive studies	[48]

**Table 2:** Dietary antioxidants modulating inflammation and redox-sensitive signaling pathways relevant to female reproduction.

Dietary Antioxidant	Dietary Source	Redox and Inflammatory Targets	Anti-Inflammatory and Redox Actions	Reproductive Relevance	Study Type/Model (In Vitro/In Vivo)	Human Clinical/ Epidemiological Investigations	Ref.
Resveratrol	Grapes, berries, red wine	NF-κB, Nrf2, SIRT1, AMPK	Suppresses NF-κB cytokine signaling, activating Nrf2 antioxidant response	Improves ovarian microenvironment, supports oocyte competence in oxidative stress	In vitro (granulosa/endothelial cells), In vivo (rodent models	Limited small clinical fertility studies; no large RCTs	[55]
Quercetin	Onions, apples, citrus	NF-κB, MAPKs (ERK, JNK, p38), Nrf2	Reduces TNF-α and IL-6, limits MAPK-driven inflammation, enhances antioxidant enzymes	Mitigates inflammatory stress affecting folliculogenesis and endometrial receptivity	Primarily in vitro, some animal models	Limited direct human reproductive trials	[14]
Epigallocatechin gallate	Green tea	NF-κB, MAPKs, Nrf2	Decreases COX-2 and pro-inflammatory mediators, strengthens antioxidant defenses	Supports biology implantation and reduces oxidative inflammation in reproductive tissues	In vitro and rodent models	Observational associations; limited fertility-specific RCTs	[56]
Curcumin	Turmeric	NF-κB, Nrf2, STAT3, MAPKs	Inhibits NF-κB and COX-2 signaling, activates Nrf2, reduces inflammatory cytokines	Relevant for endometriosis-associated inflammation and fertility impairment	In vitro and in vivo animal models	Limited small-scale fertility and PCOS clinical data	[57]
Sulforaphane	Broccoli sprouts, cruciferous vegetables	Nrf2, Keap1, NF-κB	Strong Nrf2 activator, enhances phase II detox enzymes, suppresses NF-κB activation	Protects against oxidative toxicant exposure impacting ovarian and endometrial function	In vitro and animal toxicology models	Limited epidemiological evidence; few controlled fertility studies	[58]
Lycopene	Tomato, watermelon	NF-κB, oxidative lipid signaling	Reduces lipid peroxidation, downregulates inflammatory mediators	May improve oxidative inflammatory status linked to PCOS and reproductive aging	Primarily in vivo animal models	Some observational studies in PCOS/metabolic health	[45]
Anthocyanins	Berries, purple grapes, purple cabbage	NF-κB, Nrf2, MAPKs	Suppress inflammatory cascades and strengthen antioxidant gene expression	Supports ovarian function, may reduce inflammatory burden affecting fertility	In vitro and animal models	Limited epidemiological fertility associations	[59]
Omega-3 fatty acids	Fatty fish, flaxseed, chia	NF-κB, eicosanoid pathways	Shifts eicosanoid profile toward pro-resolving mediators, reduces cytokine signaling	Improves metabolic inflammation in PCOS and supports pregnancy immune balance	In vivo models	Multiple clinical trials in PCOS and reproductive outcomes	[60]
Extra-virgin olive oil polyphenols	Olive oil	NF-κB, Nrf2, cytokine signaling	Decreases inflammatory mediator expression and promotes antioxidant defenses	Supports endometrial health and systemic metabolic-inflammatory homeostasis	In vitro and animal models	Epidemiological evidence within Mediterranean diet studies	[61]
Gingerols (ginger)	Ginger	NF-κB, COX-2, MAPKs	Inhibits COX-2 and inflammatory cytokines, reduces oxidative stress signaling	Potential benefits in inflammatory reproductive conditions and implantation stress	Mainly in vitro and animal models	Very limited human reproductive data	[62]

**Table 3:** Dietary antioxidants reported to modulate autophagy in female reproduction.

Dietary Antioxidant	Major Dietary Source	Autophagy Targets and Markers	Autophagy Effect	Reproductive Relevance and Context	Study Type/Model (In Vitro/In Vivo)	Human Clinical/ Epidemiological Investigations	Ref.
Resveratrol	Grapes, berries, peanuts	AMPK, SIRT1, mTOR, LC3-II, Beclin-1, p62	Promotes protective autophagy, supports mitochondrial quality control	Oocyte quality, ovarian aging, oxidative stress protection in ovarian cells	In vitro (ovarian cells), In vivo (rodent ovarian aging models)	Limited small clinical fertility studies; no large RCTs	[65]
Curcumin	Turmeric	AMPK, PI3K/AKT/mTOR, LC3, Beclin-1, p62	Restores autophagic flux, limits inflammation-driven damage	Endometriosis-like inflammation, ovarian stress, follicular survival support	In vitro and animal models	Limited clinical data in PCOS/endometriosis; no large ART trials	[66]
Epigallocatechin gallate	Green tea	AMPK, mTOR, MAPKs, LC3, p62	Enhance stress-adaptive autophagy, reduces ROS-linked injury	Oocyte maturation support under oxidative stress, endometrial cellular resilience	In vitro and rodent models	Observational dietary associations; limited fertility-specific RCTs	[67]
Quercetin	Onion, apple, citrus	AMPK, mTOR, LC3, Beclin-1, Bax/Bcl-2 crosstalk	Normalizes dysregulated autophagy, reduces oxidative apoptosis	Follicular function and granulosa cell protection in inflammatory stress	Primarily in vitro; some animal inflammation models	Very limited reproductive clinical studies	[68]
Berberine	Barberry, goldenseal (nutraceutical use common)	AMPK, mTOR, ULK1, LC3, p62	Improves autophagy and mitophagy, supports metabolic homeostasis	PCOS-related metabolic stress, ovarian function support via AMPK activation	In vitro and in vivo PCOS models	Clinical trials in PCOS and metabolic infertility	[69]
Melatonin	Present in some foods, endogenous	Mitophagy regulators, mPTP, LC3, PINK1/Parkin (reported)	Enhances mitophagy, preserves mitochondria, reduces ROS	Oocyte competence, embryo development support, ovarian oxidative injury reduction	In vitro (oocyte models) and in vivo animal studies	Clinical studies in IVF patients showing improved oocyte quality	[70]
Sulforaphane	Broccoli sprouts, crucifers	Nrf2, Keap1, AMPK, LC3, p62	Coordinates antioxidant defense with autophagy regulation	Protects ovarian and endometrial cells from oxidative and toxicant stress	In vitro and toxicology animal models	Limited epidemiological evidence; no large fertility RCTs	[71]
Genistein	Soy, legumes	PI3K/AKT/mTOR, ER signaling crosstalk, LC3	Modulates autophagy in hormone responsive contexts	Endometrial biology and endocrine-linked oxidative stress conditions	In vitro and animal endocrine models	Observational studies in soy-rich diets and reproductive health	[72]
Spermidine	Wheat germ, soy, mushrooms, aged cheese	EP300 inhibition (reported), autophagy induction, LC3	Induces autophagy, supports cellular housekeeping	Reproductive aging models, improves cellular stress tolerance in reproductive tissues	In vitro and lifespan animal models	Limited human aging studies; no specific reproductive RCTs	[73]
Omega-3 fatty acids	Fatty fish, flaxseed, chia	AMPK, mTOR, inflammatory lipid mediators, LC3	Supports balanced autophagy and reduces inflammatory stress	PCOS metabolic inflammation, pregnancy-related inflammatory balance, ovarian protection	In vivo animal models	Multiple clinical trials in PCOS and fertility outcomes	[74]

**Table 4:** Dietary antioxidants reported to induce apoptosis in female reproductive conditions.

Dietary Antioxidant	Dietary Sources	Female Reproductive Condition, Model	Apoptosis-Related Mechanisms	Study Type/Model (In Vitro/In Vivo)	Human Clinical/Epidemiological Investigations	Ref.
Resveratrol	Grapes, berries, peanuts	Endometrial cancer, in vitro	Increased sub-G1 fraction, Bax upregulation, caspase-3 activation, Bcl-2 downregulation	In vitro (endometrial cancer cell lines)	No direct clinical trials in endometrial cancer	[76]
Quercetin	Onions, apples, citrus	Ovarian carcinoma, in vitro	Extrinsic and intrinsic apoptosis activation, death receptor and mitochondrial pathways, caspase involvement	In vitro (ovarian cancer cell lines)	No specific human fertility or oncology RCTs; limited observational evidence	[77]
Curcumin	Turmeric	Endometriosis, in vivo and lesion tissue context	Regression of endometriosis with enhanced apoptosis in endometriomas and inhibition of inflammatory signaling (NF-κB)	In vivo (animal models) and lesion tissue analysis	Limited small clinical studies in endometriosis; not standardized therapy	[78]
EGCG (epigallocatechin-3-gallate)	Green tea	Endometrial cancer, Ishikawa cells and primary adenocarcinoma cells	Annexin V/PI apoptosis induction, anti-proliferative activity with apoptosis readouts	In vitro (cancer cells)	Epidemiological associations with green tea intake	[79]
Genistein	Soy foods	Ovarian cancer, BG-1 variants	Induces apoptosis (reported caspase-8 dependent pathway in specific settings), ER-related effects	In vitro (ovarian cancer models)	Observational studies in soy consumption and reproductive health	[80]
Sulforaphane	Broccoli sprouts, cruciferous vegetables	Endometrial cancer, cell lines and preclinical evaluation	Mitochondrial-mediated apoptosis, with pathway links to AKT/mTOR and stress signaling	In vitro and preclinical in vivo models	Limited epidemiological evidence for cruciferous intake; no targeted RCTs in endometrial cancer	[81]
Apigenin	Parsley, celery, chamomile	Endometriosis, human endometriosis cell lines	ROS-dependent apoptosis, mitochondrial membrane potential disruption, Bax and cytochrome-c changes	In vitro	No human interventional trials in endometriosis	[82]
Luteolin	Celery, green pepper, herbs	Cervical cancer (HPV-associated), in vitro	Induces apoptosis via intrinsic and extrinsic pathways, caspase-3 and caspase-8 activation, E6/E7 suppression	In vitro	No specific cervical cancer clinical supplementation trials	[83]
Anthocyanin (Cyanidin-3-glucoside, C3G)	Berries, purple grapes, purple cabbage	Ovarian cancer, in vitro and in vivo	Growth inhibition with apoptosis-related effects in ovarian cancer models	In vitro and in vivo (animal cancer models)	Limited observational dietary data; no RCTs	[84]
Lycopene	Tomatoes, watermelon	Ovarian oxidative injury/follicular reserve impairment, preclinical	Reduced ovarian damage with changes consistent with lowered apoptotic signaling, including caspase-3–positive cells reported	In vivo (preclinical ovarian injury models)	Observational associations in reproductive aging and PCOS; limited interventional fertility data	[85]

**Table 5:** Dietary antioxidants in hormonal regulation and endocrine interactions related to female reproduction.

Dietary Antioxidant	Major Dietary Source	Endocrine Targets and Pathways	Main Hormonal and Metabolic Actions	Reproductive Relevance	Study Type/Model (In Vitro/In Vivo)	Human Clinical/Epidemiological Investigations	Ref.
Resveratrol	Grapes, berries, peanuts	SIRT1, AMPK, aromatase regulation, insulin signaling	Enhances insulin sensitivity and regulates steroidogenesis-related signaling.	Facilitates ovulatory activity under metabolic stress, pertinent to PCOS and reproductive aging.	In vitro and in vivo animal models	Randomized clinical trials in PCOS showing improved insulin and androgen profiles	[90]
Quercetin	Onions, apples, citrus	PI3K/AKT, AMPK, inflammatory hormone crosstalk	Mitigates oxidative inflammation that impairs gonadotropin responsiveness	May enhance the follicular milieu and hormonal response	In vitro and animal models	Limited clinical trials in PCOS and metabolic parameters	[91]
Epigallocatechin gallate	Green tea	AMP-activated protein kinase, insulin signaling pathways, androgen-related pathways	Enhances metabolic indicators associated with hyperandrogenism	Possible advantage for endocrine dysregulation associated with PCOS	In vitro and in vivo rodent PCOS models	Some human interventional studies in PCOS	[92]
Curcumin	Turmeric	NF-κB, insulin-related pathways, steroidogenic enzymes	Reduces inflammatory signals and promotes metabolic hormone equilibrium.	May enhance ovarian steroidogenesis and menstrual cycle regularity in inflammatory conditions.	In vitro, in vivo animal studies	Clinical trials in PCOS demonstrating improved metabolic markers	[93]
Omega-3 fatty acids	Fatty fish, flaxseed, chia	Eicosanoid pathways, insulin signaling, adipokines	Enhances adiponectin and inflammatory lipid mediators, promotes metabolic endocrine equilibrium	Facilitates ovulatory function and maintains immune-endocrine homeostasis during pregnancy	In vivo animal studies	Multiple RCTs in PCOS and fertility outcomes	[94]
Genistein	Soy, legumes	Estrogen receptors (ERα/ERβ), endocrine modulation	Phytoestrogen activity influences estrogen receptor signaling and gene expression.	Pertinent to endometrial function and menopausal symptoms, necessitates dose-dependent interpretation.	In vitro and animal endocrine models	Observational studies and limited interventional trials	[95]
Lignans (e.g., secoisolariciresinol)	Flaxseed, sesame	Estrogen metabolism, SHBG modulation (reported)	Affects estrogen metabolism and the binding of circulating hormones	May facilitate hormonal equilibrium throughout reproductive age and menopause.	Mainly in vivo dietary models	Epidemiological associations in menopausal women	[96]
Coenzyme Q10	Meat, fish, whole grains	Mitochondrial steroidogenic support, ovarian energetics	Facilitates ATP-dependent steroidogenesis and mitochondrial activity	May enhance ovarian reserve indicators and oocyte viability in aged	In vivo animal models	RCTs in diminished ovarian reserve and IVF patients	[97]
Vitamin D	Fatty fish, fortified foods, sunlight	VDR signaling, AMH, insulin sensitivity, inflammation	Regulates endocrine and immune signals, enhances metabolic profile	Linked to ovarian reserve indicators and metabolic characteristics of PCOS	In vitro and in vivo studies	Large epidemiological studies and clinical supplementation trials	[98]
Myo-inositol	Fruits, beans, grains	Insulin signaling, FSH signaling, oocyte maturation	Enhances insulin sensitivity and ovarian responsiveness	Frequently utilized in polycystic ovary syndrome to enhance ovulation and hormonal equilibrium.	In vivo metabolic models	Multiple RCTs in PCOS and ART outcomes	[99]

**Table 6:** Clinical evidence for dietary antioxidants in alleviating menopause-related metabolic dysfunction and age-associated reproductive decline.

Dietary Antioxidant/Intervention	Typical Clinical Population	Key Endpoints Reported	Main Findings Relevant to Menopause and Metabolic Dysfunction	Study Type/Model (In Vitro/In Vivo)	Human Clinical/ Epidemiological Investigations	Ref.
**Resveratrol** (often with **vitamin C**)	Postmenopausal women	Oxidative stress biomarkers, insulin resistance	Reduced oxidative stress, studies also target insulin resistance and cardiometabolic risk	Primarily in vivo clinical supplementation	Randomized clinical trials in postmenopausal cohorts	[116]
**Vitamin C**	Postmenopausal women (often combined)	Total antioxidant capacity, oxidative stress	Used as redox support, commonly paired with polyphenols in interventions	Clinical supplementation	Observational and interventional studies	[116]
**Vitamin E**	Menopausal women	Lipid profile, menopausal outcomes	Results are mixed: some trials report limited lipid effects, broader reviews discuss vascular and symptom outcomes	Clinical supplementation studies	RCTs and meta-analyses in menopausal women	[117]
**Omega-3 fatty acids** (fish oil)	Postmenopausal women	Triglycerides, HDL, LDL	Reduced triglycerides with modest lipid changes overall, supports inflammation-lipid axis	In vivo and clinical trials	Multiple RCTs in postmenopausal and metabolic syndrome populations	[118]
**Coenzyme Q10**	Metabolic risk groups, also women focused cohorts	Adipokines, inflammation, insulin resistance (context dependent)	Meta-analytic evidence suggests improved adipokine profiles in metabolic syndrome trials, mechanistically consistent with mitochondrial support	In vivo metabolic models	Meta-analyses and RCTs in metabolic syndrome	[119]
**Selenium + Coenzyme Q10**	Older adults with low selenium status (sex analyses available)	Cardiovascular outcomes, oxidative stress related endpoints	Long-term RCT follow-up shows reduced cardiovascular mortality, relevant to menopause cardiometabolic risk biology	In vivo clinical RCT	Long-term randomized controlled trial data	[120]
**Alpha-lipoic acid + inositol**	Postmenopausal women with metabolic syndrome features	Insulin resistance, metabolic syndrome components	Combination improved insulin sensitivity and metabolic syndrome features in postmenopausal women	In vivo clinical study	Interventional clinical trial	[121]
**Green tea extract** (catechins, EGCG-rich)	Postmenopausal women, including overweight groups	Lipids, adipose dysfunction markers	RCTs suggest improvements in lipid profile in postmenopausal women, some studies report adipose tissue related benefits	In vivo and clinical intervention	RCTs in postmenopausal women	[122]
**Curcumin** (including enhanced formulations)	Menopausal women, metabolic risk contexts	Lipids, metabolic markers, symptoms	Trials in menopausal contexts exist, and meta-analyses evaluate postmenopausal outcomes, with growing interest in bioavailable formulations	In vivo and clinical supplementation	Clinical trials and meta-analyses	[123]
**Soy isoflavones** (genistein, daidzein)	Postmenopausal women	Lipids, triglycerides, HDL	Evidence suggests lipid benefits in pooled analyses, although older individual trials show variability	In vivo clinical supplementation	RCTs and epidemiological soy intake studies	[124]
**Flaxseed** (lignans, ALA)	Postmenopausal women	Total cholesterol, LDL-C	RCT evidence supports lipid profile improvement in postmenopausal women	In vivo clinical trial	Randomized clinical trial evidence	[125]
**Lycopene**	Postmenopausal women	Oxidative stress markers (and related health endpoints)	Supplementation increased antioxidant capacity and reduced oxidative stress, relevant to menopause-linked aging biology	In vivo clinical study	Supplementation trials in postmenopausal cohorts	[126]
**Cocoa flavanols** (polyphenol-rich cocoa)	Adult women, including postmenopausal vascular research	Insulin resistance, vascular function, BP	Systematic reviews report improvements in vascular function and insulin resistance measures in some contexts	In vivo and clinical settings	Systematic reviews and RCTs	[127]
**Pomegranate products** (juice, extracts)	Adults with metabolic syndrome and cardiometabolic risk, includes women-focused discussions	Blood pressure, glycemic markers, insulin resistance	Meta-analyses show improvements in glycemic indices in adults, and trials report BP benefits in metabolic syndrome, relevant to menopausal cardiometabolic risk	In vivo clinical research	Meta-analyses and RCTs	[128]
**Melatonin** (diet-associated, also supplement)	Adults with metabolic syndrome (includes women)	BP, lipids, glucose, waist circumference	Pilot RCTs in metabolic syndrome populations support a role in metabolic components, relevant to menopause-related sleep-metabolic interactions	In vivo and pilot RCTs	Pilot randomized clinical trials	[129]

## Data Availability

No new data were created or analyzed in this study. Data sharing is not applicable to this article.
